# Mass Effect of Pulmonary Sequestration: Multiloculated–Multiseptated Pneumomediastinum 

**Published:** 2016-04-10

**Authors:** Sezin Unal, Canan Türkyılmaz, Betül Emine Derinkuyu, Selma Aktaş, Ebru Ergenekon, Öznur Boyunağa, Yıldız Atalay

**Affiliations:** 1 Division of Neonatology, Department of Pediatrics, Gazi University Hospital, Ankara, Turkey; 2 Department of Radiology, Gazi University Hospital, Ankara, Turkey

**Keywords:** Pneumomediastinum, Pulmonary sequestration, Neonate, Respiratory distress

## Abstract

Pulmonary sequestration (PS) and pneumomediastinum are two rare clinical diseases. Pneumomediastinum was generally observed in infants either with diseased lungs or who were performed assisted ventilation or resuscitation following birth. It was reported in patients with existing ectopic thoracic kidney and laryngeal cysts however, no coexisting congenital lung anomalies were reported. Here, we report the pneumomediastinum occurred due the extralobar PS because of the mass effect of the lesion.

## CASE REPORT

Term baby boy was delivered by elective cesarean section from a 25-year-old primigravida who was routinely followed and had an uneventful pregnancy. Mother had no history of infection. Birth weight was 3660 grams and the APGAR scores were 9 at 1 minute and 10 at 5 minutes. He did not require resuscitation; just gentle nasal suction was performed. He was admitted to neonatal intensive care unit (NICU) at 15th minutes with severe respiratory distress including tachypnea (respiratory rate: 98 breaths/min), subcostal retractions, nasal flaring and hypoxia (preductal SaO2: 78%, postductal SaO2: 80%). Baby was put on oxyhood with a requirement of fractional inspired oxygen of 80%. Supine chest radiograph showed typical “spinnaker sail sign” of pneumomediastinum with bubbled appearance of air (Fig. 1a) which was also confirmed with cross table lateral views (Fig. 1b). No pneumothorax was seen. Umbilical artery and venous catheterizations were performed. Arterial blood gas analysis showed normocarbia with hypoxemia. C-reactive protein (CRP) level was 19 mg/dl for which the normal range was up to 6 mg/dl in our local laboratory. Intravenous ampicillin and amikacin were started for empirical therapy. Chest radiogram at the next day showed a new appearance resembling infiltration on left basal lobe. Computed tomography (CT) of thorax with contrast demonstrated multiloculated-multiseptated 45x45x12 mm air collection in the anterior mediastinum located between the thymus superiorly and the heart inferiorly (Fig. 2a). Moreover, there were infiltrations in the inferior lobes of bilateral lungs and left basal extralobar PS with arterial supply originating from thoracic aorta (Fig. 2b). Antibiotic therapy was switched to vancomycin and piperacillin-tazobactam due to infiltrations and persistently high CRP as 15 mg/dl. Unfortunately venous drainage could not clearly be visualized because of umbilical artery catheter that was causing artefact. But a suspicious vascular structure was noticed parallel to the systemic feeding artery (Fig. 2c) which was confirmed as a draining vein on Doppler ultrasound. Besides, the vein of the PS was draining into the azygos vein. Blood cultures were sterile, CRP level were negative in the 10th day of treatment, antibiotic therapy continued for 10 days. He required oxygen support for 17 days and was discharged at the end of third week. At the one-month follow-up, his vital signs and physical examination were normal, and well growing with exclusive breastfeeding. 

**Figure:**
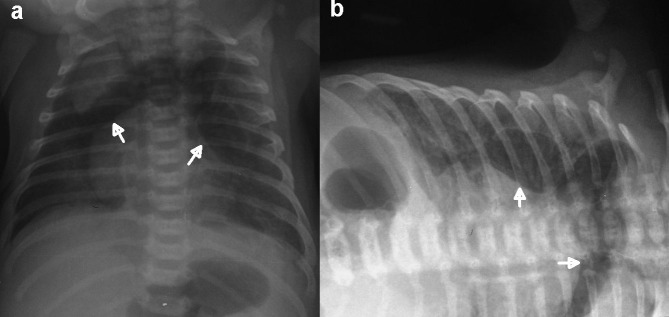
Figure 1: Chest X-ray films: (a) supine position (b) cross-table lateral view. Chest X-ray films demonstrate multiloculated large quantity of air in the mediastinum (white arrows), causing elevation of the thymus. There is no displacement of the multiseptated- multiloculated air on cross table lateral view.

**Figure:**
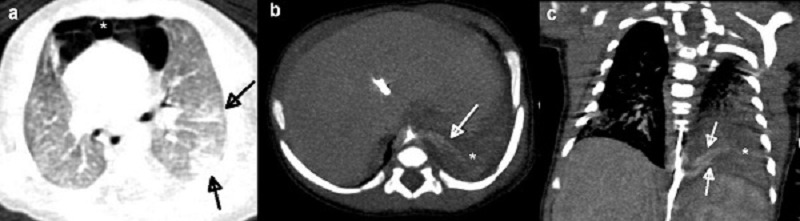
Figure 2: Contrast enhanced chest CT images of the newborn. (a) Axial plane CT in lung window reveals multiloculated, multiseptated, anterior mediastinal air collection between the pericardial sac and the thymus compatible with pneumomediastinum (asterix). Particularly, there are some ground glass opacities and reticulonodular opacities indicating an infectious process in the inferior lobe of the left lung (black arrows). Axial plane reconstructed Chest CT in mediastinal window (b), demonstrates the anomalous mass like tissue which is the pulmonary sequestration located in the left lower lobe (asterix). It has a systemic arterial supply which is a branch of the aorta (white arrow). On coronal view (c), note the pulmonary sequestration (asterix) and other vascular structure parallel to the artery (white arrow) which is a draining vein to the azygos vein confirmed by Doppler sonography.

## DISCUSSION

PS may have a role in developing of pneumomediastinum in a way that producing uneven entry of air by mass effect as it is an extralobar PS with a distinct pleural covering giving rise to complete anatomical separation from adjacent lung.[1]

Typical radiographic feature of pneumomediastinum was first described by Moseley as “spinnaker-sail sign” due to elevation of thymus upward and laterally by air accumulation in the mediastinum.[2] Pathogenesis of pneumomediastinum was explained by “Macklin effect” which is the centripetal dissection of released air from ruptured alveoli defining the passage of air through perivascular spaces of the lung, to the hilum and then to areolar tissue of mediastinum, ending up with pneumomediastinum.[3] In literature few cases presented with multiloculated-multiseptated pneumomediastinum resulting in a distinctive “bubbly” appearance on chest radiographs as seen in our patient.[4, 5] This “bubbly” appearance can be explained by findings of Quattromani et al.[6] Authors observed that thymic fascia was also continuous with the fascia of the great vessels, trachea, fibrous pericardium, and pulmonary vessels at hilum.[6] When air dissects within fascia, the “bubbly” appearance occur. The interesting aspect of our case is that multiseptated and multiloculated pneumomediastinum was occurred soon after birth without positive pressure ventilation, which can be due to ventilation inhomogeneity that was caused by mass effect of PS.[5] 

It is hard to know the exact etiology of severe respiratory distress in our patient; pneumomediastinum, PS or probable pneumonia; which was challenging during follow up. Treatment of pneumomediastinum in neonates depends up on severity of respiratory distress and development of complications such as a tension pneumomediastinum with tamponade, concomitant tension pneumothorax and/or pneumopericardium. Low et al performed a curative surgery and excised the lesion because of ongoing respiratory distress in a 3 day old term infant.[5] The pneumomediastinum in our patient was well localized to anterior mediastinum. It caused a slight pressure to left basal lobe without signs of tension pneumomediastinum. Besides, it was multiseptated and we decided that mediastinal tube drainage not to be beneficial to resolve respiratory symptoms as performed in other non-septated cases.[7] But in our patient despite spontaneous resolution of pneumomediastinum, confirmed with chest radiographs, with oxygen treatment, respiratory distress still persisted so accompanying PS or pneumonia might be the causative etiology. As clearly known congenital pneumonia may lead to prolonged respiratory distress symptoms. Underlying congenital pneumonia could not be excluded as our case had persistently high CRP levels and bilateral basal consolidation areas which were found on thoracic CT scan. 

Asymptomatic PS may not require surgery at all.[8, 9] We are following our patient and in case of respiratory infections or distress we will go for surgical excision of the PS.

## Footnotes

**Source of Support:** Nil

**Conflict of Interest:** Nil
